# Breakthroughs and challenges of organoid models for assessing cancer immunotherapy: a cutting-edge tool for advancing personalised treatments

**DOI:** 10.1038/s41420-025-02505-w

**Published:** 2025-05-07

**Authors:** Qian Wang, Fangwei Yuan, Xianglin Zuo, Ming Li

**Affiliations:** 1 Department of Thoracic Surgery, The Affiliated Cancer Hospital of Nanjing Medical University & Jiangsu Cancer Hospital & Jiangsu Institute of Cancer Research, Jiangsu Key Laboratory of Molecular and Translational Cancer Research, Collaborative Innovation Center for Cancer Personalized Medicine, Nanjing, 210009 Jiangsu PR China; 2 The Fourth Clinical College of Nanjing Medical University, Nanjing, 210009 Jiangsu PR China; 3 Department of Thoracic Surgery, Lian Shui County People’s Hospital, Huaian, 223400 Jiangsu PR China; 4 Biobank of Jiangsu Cancer Hospital (Jiangsu Institute of Cancer Research & The Affiliated Cancer Hospital of Nanjing Medical University), Nanjing, 210000 Jiangsu PR China

**Keywords:** Cancer models, Tumour immunology

## Abstract

Organoid models are powerful tools for evaluating cancer immunotherapy that provide a more accurate representation of the tumour microenvironment (TME) and immune responses than traditional models. This review focuses on the latest advancements in organoid technologies, including immune cell co-culture, 3D bioprinting, and microfluidic systems, which enhance the modelling of TME and facilitate the assessment of immune therapies such as immune checkpoint inhibitors (ICIs), CAR-T therapies, and oncolytic viruses. Although these models have great potential in personalised cancer treatment, challenges persist in immune cell diversity, long-term culture stability, and reproducibility. Future developments integrating artificial intelligence (AI), multi-omics, and high-throughput platforms are expected to improve the predictive power of organoid models and accelerate the clinical translation of immunotherapy.

## Facts

1. Organoid models provide a three-dimensional simulation of the TME, better preserving tumour heterogeneity and aiding in the development of personalised immunotherapies.
2. ICIs and CAR-T cell therapies show unique advantages within organoid models; however, their efficacy in solid tumours remain limited by the TME.
3. Co-culture systems enable organoids to predict patients’ responses to treatment and facilitate the development of novel therapeutic strategies, highlighting its usefulness in studying immune evasion mechanisms.
4. The integration of AI and microfluidic technology is expected to accelerate the clinical translation of organoid-based therapies, positioning organoids as key tools in precision medicine.
5. Challenges in standardizing organoid models include culture stability, the accuracy of drug sensitivity testing, and limitations in immune system simulation.

## Open Questions

1. How can organoid models be further optimized to more accurately simulate complex immune networks and improve long-term culture stability?
2. Can organoids in co-culture systems help identify novel biomarkers for predicting immunotherapy efficacy?
3. Can the combination of organoid models with CAR macrophage (CAR-M) therapy effectively penetrate the TME of solid tumours and enhance anti-tumour effects?
4. How can the standardization of organoid models be achieved to improve their reproducibility and scalability in preclinical and clinical research?

## Introduction

Cancer immunotherapy is a transformative approach in cancer treatment that harnesses the body’s immune system to target and eliminate tumour cells [1, 2]. Therapies, including ICIs, adoptive cell therapies (ACTs) (e.g., CAR-T), tumour vaccines, and oncolytic viruses, have shown promising results in the treatment of various cancers. However, the efficacy of immunotherapy in solid tumours remains limited, with response rates often below 20% [3–24]. This limitation is primarily attributed to the complex TME and immune evasion mechanisms [25, 26], and insufficient predictive models for personalized treatment.

Organoids are a three-dimensional (3D) culture platform that preserves tumour heterogeneity and microenvironmental features and represent valuable tools for cancer research [27, 28]. Relative to conventional 2D cell lines or animal models, organoids more accurately reflect the biological properties of tumours and their interactions with immune components. This makes them well-suited for evaluating personalised immunotherapy strategies. However, several challenges—such as the incomplete simulation of immune system dynamics, variability in experimental protocols, and maintaining long-term stability—must be addressed to fully unlock the potential of organoid-based immunotherapy.

This review discusses the various advancements in organoid technologies, focusing on their applications in immunotherapy. It further examines the integration of immune cells with organoid models, including co-culture systems, as well as innovative approaches, such as 3D bioprinting and microfluidic organoids. We also highlight the challenges and limitations of these models, offering perspectives on how they can be optimized to better simulate immune responses and improve therapeutic outcomes in cancer immunotherapy.

## Establishment of organoid models

### Organoid models

Organoids are miniature structures derived from stem or tissue-derived cells within a 3D culture matrix [29], making them an invaluable tool for cancer research and therapeutic screening. The development of organoid technology has progressed significantly over the last two decades, driven by advances in stem cell biology and tissue engineering. Organoids were first established as self-organizing systems derived from intestinal stem cells, and their ability to replicate the complexity of human tissues quickly captured the attention of researchers. A seminal study by Sato et al. demonstrated that single Lgr5+ stem cells from the mouse intestine could generate crypt-villus structures in vitro without the need for a mesenchymal niche, providing a foundational model for organoid culture in various tissues [30]. Since then, organoids have been derived from a wide range of tissues, including the gastrointestinal tract, liver, lung, and brain, providing powerful tools for modelling human diseases, including cancer [29].

Several key studies have contributed to the establishment of organoid culture techniques for specific cancers. For instance, Sato et al. demonstrated the long-term expansion of human colon organoids derived from normal tissue, adenoma, adenocarcinoma, and Barrett’s epithelium, and provided a versatile model for colorectal cancer research [31]. Gao et al. established organoid cultures from patients with advanced prostate cancer, paving the way for personalized cancer therapy [32]. Furthermore, van de Wetering et al. developed a biobank of living colorectal cancer organoids, which has become an essential resource for cancer research and drug testing [33]. Similarly, organoids derived from liver cancer, breast cancer, and gastric cancer have enabled the study of tumour heterogeneity and the identification of therapeutic targets [34–36]. Moreover, organoids derived from other cancers, including pancreatic cancer, oral cancer, ovarian cancer, glioblastoma, have also been developed to study cancer-specific drug responses, disease progression, and heterogeneity [37–40].

A key challenge in constructing tumour organoids lies in the complexity of tumour and non-tumour cells in the cell suspension. To prevent the overgrowth of non-tumour cells, medium optimisation is essential. Optimising the culture medium is essential to ensure the growth of tumour cells while preventing the overgrowth of non-tumour cells. Specific cytokines, such as Noggin and B27, are often added to inhibit fibroblast proliferation while promoting the expansion of tumour cells. The exact culture conditions can vary depending on the tumour type, requiring the addition of multiple soluble factors to promote the growth of organoids (Table 1) [33, 41–48]. These soluble factors are biologics, including growth factors and small molecules, that can activate or inhibit specific signalling pathways to promote tumour cell growth. Moreover, growth factors such as Wnt3A and Noggin play crucial roles in the maintenance of stemness and differentiation in organoids by positively regulating the Wnt signalling pathway, and are applicable to the growth of various organoids. Therefore, researchers need to adjust the medium composition to maintain tumour organoid growth and function. For instance, HGF plays an important role in hepatocyte regeneration and proliferation but its activity is lower in other tissues; therefore, it is often not used in organoid models other than liver cancer models. Combinations of specific growth factors, cytokines, and inhibitors help organoids retain their morphology and genetic characteristics over extended periods, accurately simulating in vivo biological conditions [29, 49–51]. This optimisation strategy enables organoid models to more realistically reflect the biological properties of tumours in the body.

**Table 1:** The components in the culture medium of organoids from various tissues.

Components	LUAD	GC	CRC	HCC	PANC	BC	EOC	BLCA	ESCA
B27	+	+	+	+	+	+	+	+	+
N-2	+	+	+	+	+				
FGF							+	+	
FGF2							+	+	
FGF-7	+					+		+	
FGF-10	+					+		+	
Wnt-3a		+	+	+	+		+		+
Noggin	+	+	+	+	+	+		+	+
EGF		+	+	+	+	+		+	+
Neuregulin1						+			
HGF				+					
IGF-1							+		
R-Spondin-1	+	+	+	+	+	+	+		+
Y-27632	+		+	+		+	+	+	
A83-01	+	+	+	+	+		+	+	+
SB 202190	+		+		+	+			
Nicotinamide	+		+	+	+	+		+	+
N-acetyl-l-cysteine	+	+	+	+	+	+	+	+	+
Wnt-C59					+				
Forskolin				+					
GastrinI		+	+	+	+		+		
prostaglandin E2		+							
References(PMID)	[41]	[42]	[33]	[43]	[44]	[45]	[46]	[47]	[48]

The ECM plays a crucial role in organoid construction, providing not only physical support but also regulating cell behaviour to maintain cell fate [52]. Matrigel, extracted from Engelbreth-Holm-Swarm tumours, is a widely used ECM material that forms a 3D gel at 37 °C, and it provides a suitable environment for various cells, including neurons, cardiomyocytes, and PSC-derived organoids [53–55]. However, due to its animal origin, Matrigel demonstrates significant batch-to-batch variability in its mechanical and biochemical properties and affect experimental reproducibility [56, 57]. To overcome these limitations, researchers have developed synthetic matrix materials, such as synthetic hydrogels and gelatin methacrylate (GelMA), which provide consistent chemical compositions and physical properties for stable organoid growth [53, 58, 59]. By precisely regulating matrix stiffness and porosity, these synthetic materials improve organoid culture outcomes, which enables more stable simulation of in vivo environments.

Organoid technology has currently progressed significantly, such as co-culture model not only replicates the complex interactions between tumours and the immune system but also provides a powerful platform for studying immunotherapy [60]. Through these models, scientists can more accurately assess the efficacy of immunotherapies and identify the most effective personalised treatment strategies [41, 46, 61–70] (Table 2).

**Table 2:** Consistency between organoid-based drug sensitivity testing (DST) and clinical trials for different cancer types.

Cancer type	Sample size	Therapeutic regime	Main results	Reference
Lung cancer	36 patients	Chemotherapy, Targeted therapy	Organoid DST predicts clinical response: 84.0% sensitivity, 82.8% specificity	[61]
	103 patients	Chemotherapy	Organoid DST predicts clinical response:100% accuracy, 100% specificity	[41]
Breast cancer	35 patients	Chemotherapy, Targeted therapy, Immunotherapy	Organoid DST predicts clinical response: 82.35% sensitivity, 69.23% specificity, 76.67% accuracy	[62]
Gastrointestinal cancer	72 patients	Chemotherapy,Targeted therapy	Organoid DST predicts clinical response: 100% sensitivity, 93% specificity, 88% positive predictive value and 100% negative predictive value	[63]
	11 patients	Chemotherapy,Targeted therapy	Organoid DST predicts clinical response: 82% concordance rate	[64]
Gastric cancer	73 patients	Chemotherapy	Organoid DST predicts clinical response: 91.7% concordance rate	[65]
Pancreatic cancer	31 patients	Chemotherapy	Organoid DST divides patients into three groups: sensitive, intermediate, and resistant, with statistical differences in PFS between the groups	[66]
	16 patients	Chemotherapy	Organoid prediction model allows a successful response prediction in treatment-naïve patients with an accuracy of 91.1% for first-line and 80.0% for second-line regimens	[67]
Pancreatic ductal adenocarcinoma	12 organoids	Chemotherapy	A method for classifying PDOs as sensitive or resistant to chemotherapy regimens was developed to predict the clinical outcome of patients.	[68]
	21 organoids	Chemotherapy	Organoid DST responses were not different from patient tumour responses	[69]
Rectal cancer	7 patients	Chemotherapy	AUC for both 5-FU and FOLFOX ex vivo treatments correlated with PFS of the corresponding patient.	[70]
Ovarian Cancer	5 patients	Chemotherapy	These PDO drug responses showed a statistically significant correlation (*p* < 0.01) with clinical response, as measured by histopathological (chemotherapy response score [CRS]), biochemical (normalisation of the serum biomarker CA-125), and radiological (RECIST) responses.	[46]

### Organoid-immune co-culture models

**Fig. 1:**
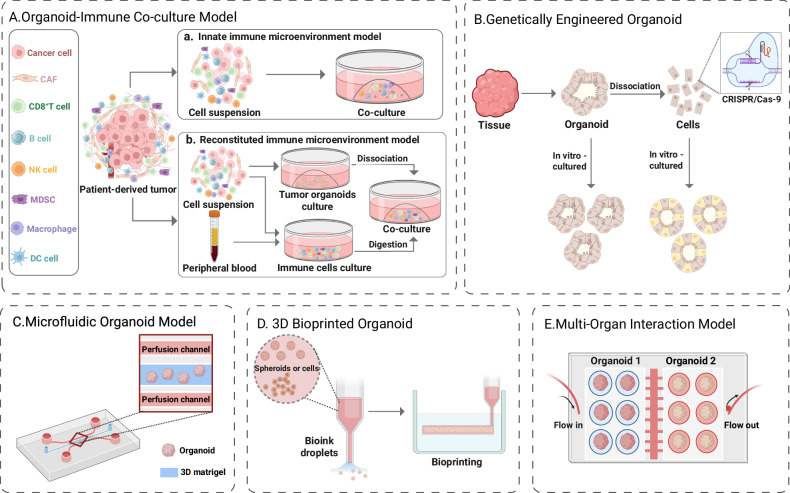
Schematic representations of different types of organoid models. **A** Organoid-immune cell co-culture model schematic, illustrating the integration of immune cells and tumour organoids to simulate the TME and study immune responses. **B** Schematic of genetically engineered organoid models, depicting the incorporation of specific genetic modifications to study the impact of mutations or therapeutic interventions on tumour behaviour and treatment responses. **C** Microfluidic organoid chip model schematic, showing the use of microfluidic technology to control environmental parameters, such as nutrient flow, oxygen gradients, and mechanical stress, for drug testing and disease modelling. **D** 3D bioprinted organoid model schematic, demonstrating how 3D printing techniques are employed to create organoids with precise structural organization and to replicate tumour architectures for more accurate disease modelling. **E** Multi-organ interaction model schematic, illustrating the integration of multiple organoid systems to study the complex interactions between different organ types. This figure was created using BioRender.com.

Organoid-immune co-culture models have emerged as powerful tools for studying the TME and evaluating immunotherapy responses. These models can be broadly categorized into innate immune microenvironment models and reconstituted immune microenvironment models, depending on the source of the immune components used in the co-culture (Fig. 1). Herein, we discuss both categories in detail and provide examples of key studies that have utilized these models.

#### Innate immune microenvironment models

In this category, organoid models are derived from tumour tissue and retain the complex structure of the TME, including immune cells within the tumour. Neal et al. developed a tumour tissue-derived organoid model that employed a liquid-gas interface, which retained the complexity of the TME [60]. These organoids maintained functional tumour-infiltrating lymphocytes (TILs) and could replicate the PD-1/PD-L1 immune checkpoint function, making them an excellent platform for studying immune responses in cancer. Jenkins RW, et al. developed murine- and patient-derived organotypic tumour spheroids (MDOTS/PDOTS) to evaluate immune checkpoint blockade (ICB) responses [71]. These models maintain autologous immune cells and enable ex vivo testing in 3D microfluidic culture. Profiling revealed that TBK1/IKKε inhibition enhanced PD-1 blockade response and predicted tumour outcomes, providing a novel platform for ICB testing.

Similarly, Voabil et al. established a tumour tissue-derived organoid platform using 1 mm³ fragments from freshly sampled tumours [72]. These tumour organoids were treated with a PD-1 inhibitor to investigate immune responses in different tumour types. Tumours with a high tumour mutational burden (TMB), such as melanoma and NSCLC, exhibited a robust immune response, which was correlated with clinical outcomes. This ex vivo model showed significant potential for predicting the efficacy of immunotherapy. To facilitate better clinical translation of this technology, Ding et al. developed a droplet-based microfluidic technology with temperature control [73]. This system allows the generation of numerous small organoid spheres from minimal tumour tissue samples, preserving the TME, and enables drug response evaluations within 14 days, offering the potential for precision tumour therapy in clinical settings.

#### Immune reconstitution models

In contrast to the previous studies, Dijkstra et al. established a co-culture model of tumour organoids and autologous peripheral blood lymphocytes [74]. The study showed that, after co-culturing with tumour organoids, the CD8+ T cell population and its cytotoxic factors significantly increased. Furthermore, the autologous reactive CD8+ T cells obtained after co-culture specifically targeted and killed tumour cells. This model allows for the separation of tumour organoids and immune cells, and can be used to assess the sensitivity of tumour cells to T cell-mediated attack. Moreover, this co-culture model is highly valuable for studying tumours with poor immune infiltration, such as pancreatic ductal adenocarcinoma (PDAC). To reproduce immune infiltration in an immunosuppressive TME, Zhou et al. encapsulated T cells in an external Matrigel layer after constructing tumour organoids [75]. The T cells from the Matrigel layer gradually infiltrated into the organoids, forming T cell-bound tumour organoids. The establishment of this model will accelerate the discovery of novel drugs that can be combined with ICIs for PDAC and may lead to the development of new precision immunotherapies in the future. This also suggests that patient-derived organoid (PDO) models involving immune cells will serve as a promising preclinical drug screening platform.

### 3D bioprinted organoid models

The use of 3D bioprinting has emerged as a powerful technology for engineering organoids by creating heterogeneous cellular microenvironments, which helps to replicate complex structures and functions of human organs. This approach offers several advantages, including precise control over cell placement, the ability to form large cellular aggregates, and improved reproducibility and standardization [76]. For example, Choi et al. developed a 3D bioprinted lung cancer organoid model that incorporated fibrotic niches using idiopathic pulmonary fibrosis-derived lung fibroblasts and LudECM. They showed that fibrosis significantly enhanced drug resistance and altered the efficacy of targeted therapies and provided a more accurate preclinical platform for assessing personalised cancer treatment [77]. Yu et al. used a 3D bioprinted PDO model to investigate the role of NSUN6 in cervical cancer radiosensitivity [78]. The study showed that higher NSUN6 expression was associated with radioresistance, and silencing NSUN6 in the PDO model enhanced radiosensitivity both in vitro and in vivo, suggesting that 3D PDO models are valuable tools for studying radiotherapy resistance and identifying therapeutic targets. Li et al. proposed a novel approach for identifying genotoxic impurities (GTIs) by using an in vitro nucleoside incubation model to generate DNA adduct profiles, followed by the verification of genotoxicity in 3D bioprinted human liver organoids [79]. The study demonstrated that the Lanchlor compound, a potential genotoxic impurity in lansoprazole synthesis, generated diverse DNA adducts. The 3D liver organoids also showed results that were similar to those obtained from rat models, confirming the utility of this combined approach for rapid GTI screening.

### Other organoid models

Other organoid models, such as microfluidic organoid chip models, genetically engineered organoid models, and multi-organ interaction models, have also gained significant attention and are increasingly being applied in various fields. These advanced models provide more dynamic and comprehensive platforms for drug testing, disease modelling, and personalized medicine. A summary of these models, along with their applications, advantages, and limitations is shown in Table 3 [80–88].

**Table 3:** Summary of advanced organoid models and their applications.

Organoid model	Description	Applications	Advantages	Challenges	References
Organoid-immune cell co-culture models	Organoid models co-cultured with immune cells to simulate tumour-immune interactions.	Immunotherapy testing, cancer immunology, immune checkpoint studies.	Replicates complex TME with immune responses, predictive of therapy outcomes.	Complexity of immune cell integration and stability.	[80, 81]
3D bioprinted organoids	3D bioprinting technology used to construct organoids with precise cell deposition.	Drug screening, disease modelling, personalized medicine.	High reproducibility, creates complex structures, automation potential.	Viability of printed cells, limited by material options and cost.	[82, 83]
Microfluidic organoid models	Microfluidic systems simulate fluid dynamics and organ-specific conditions in organoids.	High-throughput drug testing, personalized medicine, toxicology screening.	Precise control of microenvironments, enables high-throughput and dynamic studies.	Complexity of chip fabrication, long-term culture stability issues.	[84, 85]
Genetically engineered organoids	Organoids modified using gene-editing technologies to model specific genetic mutations.	Cancer research, drug resistance studies, disease modelling.	Precision in modelling genetic mutations, enables personalized therapy testing.	Gene editing efficiency and stability, off-target effects.	[86, 87]
Multi-organ interaction models	Integration of multiple organoid types to simulate inter-organ interactions and systemic diseases.	Systemic disease modelling, drug metabolism, immune response studies.	Simulates organ-organ interactions, useful for studying multi-organ diseases.	High complexity, integration of different organoid types is challenging.	[84, 88]

## Applications of organoid models in cancer immunotherapy

Immunotherapy, including ICIs, ACTs (e.g., CAR-T, TCR-T), tumour vaccines, and oncolytic viruses (Table 4) [89–102], has revolutionized cancer treatment by harnessing the immune system to target tumours. Despite these advancements, the efficacy of such therapies is often limited by tumour immune evasion mechanisms, such as the TME and antigen heterogeneity. These challenges underscore the need for more sophisticated models to better optimize and personalize therapeutic strategies.

**Table 4:** Current immunotherapies: mechanisms, clinical applications.

Therapy	Mechanism	Key agents	Clinical applications	Key challenges	Reference(PMID)
Immune checkpoint inhibitors (ICIs)	Block PD-1/PD-L1 or CTLA-4 to restore T-cell activity	Ipilimumab Nivolumab Pembrolizumab Atezolizumab	Melanoma, NSCLC, RCC, Hodgkin lymphoma etc.	Lack of reliable biomarkers; drug resistance; low response rates in solid tumours	[89–92]
CAR-T therapy	Genetically engineered T cells targeting tumour antigens	Kymriah Yescarta	B-cell ALL, DLBCL	Poor infiltration in solid tumours; antigen escape; TME suppression	[93, 94]
Tumour vaccines	Activate APCs to prime tumour-specific T cells	Provenge	Prostate cancer	Immunosuppressive TME; antigen loss; limited durability	[95, 96]
Oncolytic viruses	Selective tumour lysis and antigen release to activate systemic immunity	T-VEC	Melanoma	Host immune clearance; ECM barriers; limited viral spread	[97, 98]
CAR-M therapy	CAR-engineered macrophages to phagocytose tumours and remodel TME	CT-0508	NCT04660929	Limited Clinical data; TME suppression	[99, 100]
Bispecific T-cell engager	Bind tumour-specific antigens on cancer cells and receptors on T cells	Glofitamab Blinatumomab Epcoritamab	NCT06656221 NCT06645678 NCT06672705	Limited efficacy in solid tumours	[101, 102]

### Role of organoids in the study of ICIs

Organoid models provide a highly realistic in vitro platform for examining the mechanisms of action of ICIs and their combination therapies. By accurately simulating the complex interactions between tumours and the immune system, these models have become essential tools for evaluating the efficacy of ICIs. Liu et al. established an automated platform combining single-cell analysis with organoids, and performed single-cell sequencing on organoids treated with ICIs [80]. The study revealed that the proportion of CD8+ T cells in the organoids was consistent with flow cytometry results. Based on immune infiltration, the samples were classified into three categories: immune desert tumours, cold tumours (with T cells outside the tumour), and hot tumours (with T cells inside the tumour). The results were consistent with Haematoxylin and Eosin (HE) staining and immunohistochemistry (IHC) validation. Moreover, after drug treatment, the survival index of hot tumours significantly increased. Further analysis identified CD99 as a key regulatory factor influencing the activation of CD8+ T cells in anti-tumour immune responses. Additionally, the organoid immunotherapy model can be used to identify biomarkers and potential therapeutic targets related to immune treatment response. Ballerini developed an “intestinal chip” system that simulates human intestinal structure and function [81]. By analysing faecal samples from patients who responded to immunotherapy and those who did not, the study found that the non-responder group exhibited stronger activation of inflammatory signalling pathways and suppression of cellular stress responses. In contrast, faecal samples from the responder group were enriched in genes related to flagellin, a natural ligand for TLR-5. The TLR-5 signalling pathway may play a significant role in regulating intestinal inflammation, thereby providing a new target for developing microbiome-based immunotherapy strategies.

However, regarding clinical applications, ICIs alone often fails to achieve the desired effects due to the complexity of the TME. The combination with other drugs can remodel the TME and enhance the anti-tumour activity of immune therapies. Zhou et al. developed pancreatic cancer organoids carrying the OVA antigen and co-cultured them with OT-I mouse T-cells [75]. Their results showed that the apoptosis rate of tumour cells was approximately 10% when using ICIs alone; however, the cytotoxic effect of T-cells increased to 30% when combined with epigenetic regulators, indicating the unique value of organoid models in optimising combination therapy strategies. Chen et al. found that high expression of the CYP4F2 gene was closely associated with immune evasion in a co-culture system of NSCLC organoids and cancer-associated fibroblasts [103]. After the application of CYP4F2 small molecule inhibitors, the apoptosis rate of tumour cells increased to 14.9%. When ICIs were used in conjunction with CYP4F2 inhibitors, the apoptosis rate in the organoids further increased to 59.1%, indicating a significant enhancement of combination therapies targeting the TME.

### Organoids in adoptive cell therapy, tumour vaccines, and oncolytic virus therapy (OVT)

Organoids have emerged as invaluable models for studying various immune therapies, including ACTs such as CAR-T and TCR-T, tumour vaccines, and OVTs. These therapies aim to enhance immune responses either through re-engineering immune cells or by utilizing external agents, including viruses and vaccines, to target and kill tumour cells.

#### Adoptive cell therapy (ACT)

CAR-T therapy, which involves engineering T cells to express a receptor specific to tumour antigens, has shown tremendous promise in haematologic malignancies. Organoid models have been used to assess the efficacy and specificity of CAR-T cells. For example, Harter et al. demonstrated that CAR-T cells effectively killed tumour organoids while sparing normal tissues, indicating the potential of CAR-T cells in precision therapy [104]. Similarly, Li et al. used organoid models to evaluate the efficacy of CD70-targeting CAR-T cells in renal cancer, and observed enhanced T-cell activation and tumour cell killing [105]. While CAR-T therapy has shown great promise, it faces challenges in treating solid tumours due to immune suppression in the TME, physical barriers, and tumour heterogeneity. To overcome these challenges, CAR macrophages (CAR-M) have emerged as a potential solution. CAR-M cells offer unique advantages, particularly in their ability to penetrate solid tumours and reshape the TME. Compared to CAR-T cells, CAR-M cells have demonstrated greater efficacy in targeting and eliminating tumour cells in solid tumours. A recent study used ovarian cancer organoids to assess the targeted infiltration and killing capacity of CAR-M cells, confirming that organoid models are valuable for evaluating the therapeutic potential of CAR-M cells in solid tumours [106]. Additionally, a study by Zhang et al. highlighted that inhibiting SIRPα could enhance the anti-tumour efficacy of CAR-M cells, further underscoring the importance of organoid platforms in optimising CAR-M therapy [107].

#### Bispecific T-cell engagers (BiTEs)

In addition to CAR-T therapy, bispecific T-cell engagers (BiTEs) have offered novel treatment options for haematologic malignancies [102, 108]. T-cell engagers (BiTEs) are an innovative class of immunotherapies designed to simultaneously bind tumour-specific antigens on cancer cells and CD3 receptors on T cells, effectively bridging the two to stimulate T-cell-mediated cytotoxicity. Co-culturing tumour organoids with T cells, allows researchers to investigate how BiTEs bridge T cells and tumour cells, leading to targeted cytotoxicity [73, 109, 110]. Additionally, organoids derived from a patient’s tumour can enable personalised screening of BiTE therapies to facilitate the identification of the most effective candidates based on individual tumour profiles.

#### Tumour vaccines

Organoids have also become indispensable in the development and optimisation of tumour vaccines. These models enable researchers to evaluate immune responses to specific tumour antigens and optimise vaccine designs. Wagar et al. developed a human tonsil organoid model that retains various immune cell types, offering a more realistic in vitro system for testing tumour vaccines [111]. Kastenschmidt et al. further used this model to study the immune response after vaccination, demonstrating how organoids can help refine vaccine strategies [112]. Moreover, organoid models can facilitate personalised vaccine development, enabling researchers to test vaccines tailored to the unique tumour antigens of individual patients. This approach helps to overcome tumour heterogeneity and immune evasion mechanisms, enhancing the efficacy of vaccines in clinical settings [113, 114].

#### Oncolytic virus therapy (OVT)

OVT involves using viruses that selectively infect and kill tumour cells while stimulating an immune response. Organoid models have proven valuable for evaluating the efficacy of oncolytic viruses (OVs) and studying their interactions with the TME. For instance, Zhu et al. used glioblastoma organoids to test the Zika virus as an oncolytic agent, and found that it induced tumour cell apoptosis through the SOX2-integrin αvβ5 pathway [115]. In a Phase II clinical trial, organoid models were used to assess oncolytic viruses in patients with malignant ascites [116]. The results demonstrated that OVs inhibited peritoneal metastasis and reduced malignant ascites, with no serious adverse effects. Additionally, gene sequencing revealed that OVs enhanced immune checkpoint interactions by promoting myeloid cell differentiation and activating CD8^+^T cells.

## Limitations and challenges of organoid models

Organoid models have demonstrated marked potential for tumour immunotherapy, but challenges remain, including incomplete immune system simulation, limitations in drug sensitivity testing, standardization issues, stability concerns during long-term culture, and high costs and material availability for large-scale use.

### Inadequacies in immune system simulation

Most organoid-immune cell co-culture models focus on a single type of immune cell, such as T cells or macrophages, provide valuable insights into the role of individual immune components but failing to replicate the complex immune networks in vivo. The actual TME involves various immune cells, including T-cells, B-cells, dendritic cells, and NK cells, working together to regulate tumour growth and therapeutic response [117, 118]. The lack of immune cell diversity limits the predictive accuracy of current models in assessing long-term immunotherapy efficacy [119].

Additionally, vascular endothelial cells and ECM components play essential roles in immune responses and drug delivery. Without these, organoid models struggle to simulate in vivo dynamics, such as hypoxia, pH gradients, and mechanical stress. These factors are critical for evaluating the long-term effects of immunotherapies, especially angiogenesis inhibitors or therapies targeting hypoxia-related pathways [120].

To overcome these limitations, future models should integrate multicellular co-culture systems that include not only multiple immune cell types but also stromal and vascular elements. Recent advancements in microfluidic technology and 3D bioprinted organoids have shown great potential in addressing these challenges [82–85]. Specifically, microfluidic organoid models can simulate fluid dynamics and nutrient gradients, while 3D bioprinting allows for precise deposition of various cell types and ECM materials, creating more realistic tumour models. However, microfluidic models face limitations in scale and complexity, and 3D bioprinted organoids struggle with long-term stability and biological complexity. These challenges can be addressed by improving device precision, developing new biocompatible materials, and integrating multiple technologies. Additionally, organ-on-a-chip platforms can mimic microenvironmental conditions more accurately, offering a promising approach when integrated with organoid models [121–123].

Furthermore, the use of AI in data analysis can enhance the complexity of immune simulations by predicting interactions between immune cells and tumour components [124, 125]. AI tools can help identify key cellular interactions within the TME and optimise experimental conditions to reflect in vivo scenarios more accurately [125–127]. Combining in vitro organoid models with in vivo experiments will also be essential for validating therapeutic predictions and ensuring clinical relevance.

### Limitations of drug sensitivity testing

Drug sensitivity testing in organoid models primarily relies on tumour cell viability, but this approach has limitations. Direct drug effects on tumour cells are often difficult to distinguish from indirect effects mediated by the TME, such as interactions with immune cells, fibroblasts, or vascular endothelial cells. For instance, immune-suppressive factors secreted by MDSCs or TAMs may reduce the observed efficacy of drugs [128, 129], leading to an underestimation of their therapeutic potential. Conversely, isolated tumour cells in vitro may respond more favourably to treatment than they would in vivo, resulting in an overestimation of clinical effectiveness due to the absence of TME-mediated resistance mechanisms.

Evaluating the efficacy of immunotherapies requires more than measuring tumour cell viability alone. It also involves monitoring immune cell activity, vascular integrity, and cytokine release, which all significantly influence therapeutic outcomes [130]. However, current organoid models often lack the ability to fully replicate the complexity of these interactions, limiting their predictive power.

Co-culture models offer a promising solution by integrating immune cells and stromal components with organoids. These models simulate more realistic TMEs, allowing the assessment of immune cell infiltration, activation, and cytotoxic effects [105]. However, they still struggle with replicating the dynamic in vivo immune interactions, particularly regarding immune cell-mediated drug resistance. Using 3D bioprinted organoid models address some of these limitations by better mimicking the TME. They enable precise control over cell organization and matrix structure, improving the representation of tumour architecture. However, challenges such as ensuring long-term stability and reproducibility remain, affecting their utility for consistent drug testing. Genetically engineered organoids allow for the study of specific genetic mutations in drug responses. These models offer insights into targeted therapies, and still do not fully capture the complexity of immune cell interactions. Integrating them with immune cell co-culture could enhance their ability to simulate tumour-immune dynamics more comprehensively.

**Fig. 2:**
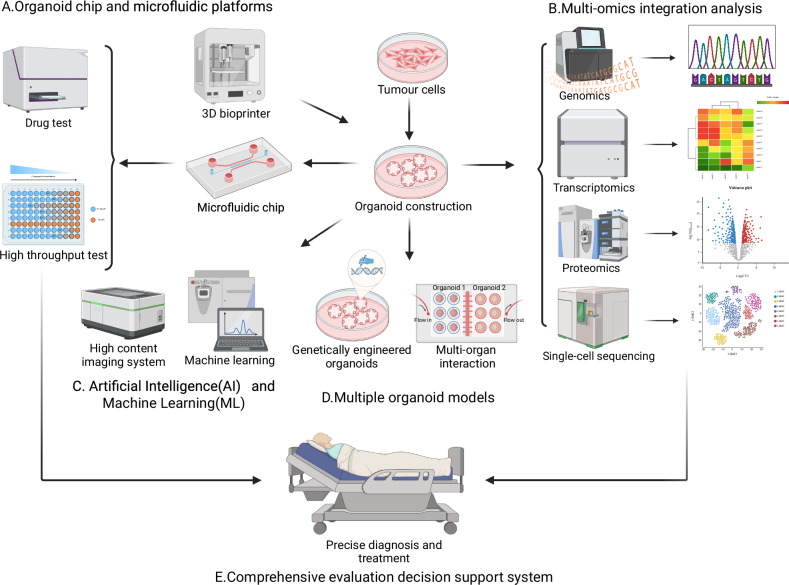
Application of tumour organoid. **A** Microfluidic technology provides a novel dynamic physiological environment for organoid research. Through microfluidic organoid chips, researchers can simulate in vivo blood flow, nutrient transport, and complex cell interactions to enable high-throughput drug screening. **B** By integrating multidimensional data, such as genomics, transcriptomics, and proteomics, it is possible to elucidate the complex molecular mechanisms involved in tumour initiation and progression. **C** The introduction of AI and machine learning provides powerful tools for the analysis and prediction of organoid experimental data. By leveraging the computational power of large AI models, the data can be deeply mined and analysed. **D** Application of gene-edited and multi-organ interaction models for cancer research. **E** Integrating organoid models with emerging technologies will facilitate the formulation of personalised treatment plans for precision oncology. This figure was created using BioRender.com.

In the future, multidimensional platforms that integrate immune responses, cytokine profiling, and real-time imaging will improve the ability to assess drug sensitivity more accurately (Fig. 2) [131–133]. AI tools, when applied to organoid-based testing, could predict drug synergies and optimize therapeutic regimens [134]. Moreover, microfluidic platforms can further refine the testing conditions by replicating fluid dynamics, nutrient gradients, and other physiological factors.

### Stability concerns in long-term culture

Achieving stable long-term culture is crucial for maintaining the functional, phenotypic, and epigenetic integrity of organoids to ensure that they accurately replicate patient-specific tumour characteristics. However, challenges such as nutrient depletion, oxygen gradients, and waste accumulation can compromise cell viability, especially in larger organoids. Frequent media changes help mitigate these issues but increase operational costs and complexity.

Synthetic hydrogels and advanced biomaterials offer promising solutions by minimizing batch-to-batch variability and providing precise control over matrix stiffness, porosity, and biochemical signals [135]. These materials support cell viability and tissue architecture, with dynamic hydrogels enhancing organoid stability by simulating ECM remodelling.

Dynamic culture systems integrated with microfluidic technology further improve stability by regulating oxygen and nutrient delivery in real time, reducing hypoxia and waste accumulation. Automated systems can further minimize manual intervention and reduce human error and variability [136].

AI tools have the potential to revolutionize long-term organoid culture. AI-driven monitoring systems can predict nutrient requirements and detect early signs of cellular stress. They also enable real-time adjustments to culture conditions. Combined with automated platforms, these technologies will not only enhance stability but also lower costs, making long-term organoid culture more feasible for large-scale applications.

### Standardization and reproducibility issues

The standardization and reproducibility of organoid models limit their broad application in preclinical and clinical settings. Variations in sample sources, culture conditions, and matrix materials contribute to inconsistent results across laboratories [56, 57]. Even organoids from the same patient can show differences in gene expression, microenvironment simulation, and drug responses. This variability complicates cross-institutional validation and reduces the reliability of results, limiting the clinical translation of organoid research.

To address these challenges, the establishment of a unified standardization framework is essential, with guidelines for sample handling, media composition, culture protocols, and data analysis to improve reproducibility. Furthermore, maintaining microenvironmental fidelity—including ECM properties and multicellular interactions—across different organoid types and experimental setups remains a key technical challenge.

Recent progress has been made toward achieving standardization. International research communities have developed consensus standards involving naming conventions, cell sources, culture techniques, validation methods, and ethical considerations. These standards apply to various organoid types, including models derived from pluripotent stem cells (such as the brain, liver, and intestinal organoids) and adult stem cells (such as the lung and prostate organoids) [137–139]. However, further optimisation is required for specific tissue-derived organoids, especially for maintaining long-term culture stability and replicating the TME with high fidelity.

Advanced matrix materials, such as synthetic hydrogels, can improve reproducibility by reducing batch-to-batch variability and offering precise control over matrix properties, porosity, and biochemical signals, which are critical for preserving organoid functionality and structural integrity. Additionally, dynamic culture systems that incorporate microfluidic technology may help better simulate physiological conditions, such as nutrient delivery and mechanical stress, and enhance reproducibility [140].

AI-driven data analysis and automated culture systems can play pivotal roles in refining organoid standardization in the future. AI-based models can optimise experimental protocols by analysing large-scale multi-omics data, predicting organoid growth patterns, and identifying key variables influencing outcomes. Automated systems, coupled with AI-guided monitoring, can minimize human error, enhance consistency, and ensure reproducibility across different research centres [141].

### Considerations of experimental material sources and costs

The construction of organoid models depends heavily on the selection and combination of various experimental materials. Co-culturing with patient-derived autologous immune cells offers the most accurate simulation of individualized treatment effects, closely reflecting the patient’s TME. However, this approach presents several operational challenges, including high costs, sample acquisition difficulties, and complex handling protocols. Additionally, biological variability between patients limits the large-scale application and reproducibility of these models.

To address these challenges, researchers have adopted commercially available cell lines as a more accessible and cost-effective alternative [118]. These cell lines offer the advantages of stability, low cost, and easy accessibility, making them popular for routine experiments. However, they often fail to replicate the specific pathological features or the complex interactions within patient-specific TME. Therefore, balancing precision and cost in material selection is critical for ensuring both the scientific validity and feasibility of organoid-based studies.

Emerging organoid models, such as 3D bioprinted organoids, microfluidic organoid models, and genetically engineered organoids, further complicate material sourcing and cost-efficiency. Although 3D bioprinted organoids offer the ability to model tumour structures more accurately by layering cells and extracellular matrix components, they require specialized equipment and advanced biomaterials, making them costly and difficult to scale for routine use. Similarly, microfluidic organoid models provide a dynamic environment that closely mimics in vivo conditions but often face challenges in terms of technical complexity and high upfront costs. Genetically engineered organoids are valuable for studying specific mutations, but necessitate precise genetic manipulation, which increases both the cost and time required to develop models.

To address the challenges related to material sourcing and costs, biobanks and shared sample repositories are being established to provide standardized patient-derived cells for easier access to diverse biological materials [142, 143]. Additionally, AI-based algorithms can aid in optimising material selection by analysing multi-omics data to identify the most representative cell lines or patient samples. Future efforts to standardize cell line selection and improve patient-derived material accessibility will enhance reproducibility, scalability, and clinical translation of organoid research, advancing personalized medicine.

## Future prospects

The future of organoid research will be shaped by key advancements that address current limitations and expand their applicability in drug testing, personalized medicine, and cancer immunotherapy.

A promising direction is the integration of AI and automation, which will enable precise predictions of patient-specific responses, particularly for immunotherapies. AI-driven multi-omics analysis will help optimize immunotherapy regimens, uncover novel immune biomarkers, and guide the development of targeted therapies [134]. Additionally, microfluidic technology and 3D bioprinting will improve model accuracy, enabling better simulation of complex tissue environments by regulating nutrient flow, oxygen gradients, and mechanical forces, which are vital for evaluating immunotherapies such as ICIs, CAR-T, and TIL therapies [41, 82, 83, 144].

Co-culture models integrating tumour organoids with immune cells, stromal components, and endothelial cells will enhance immunotherapy testing by simulating tumour-immune interactions and immune resistance. These models will help better simulate the immune evasion processes in the TME and predict responses to ICIs, CAR-T cells, and tumour vaccines. As organoid models evolve, they will increasingly incorporate PDOs and genetically engineered organoids, offering tailored approaches to predict treatment efficacy and patient-specific responses to therapies, especially in the context of immunotherapy [122, 145].

Furthermore, addressing the standardization and reproducibility of organoid models will be critical for their widespread use in clinical settings [146]. Ongoing efforts in optimizing culture protocols, data sharing, and validation methods will ensure that organoid models are consistent, reliable, and scalable for large-scale applications, particularly in clinical trials for immunotherapies [145]. As these advancements unfold, organoid-based drug testing will offer better predictive models for personalized cancer treatment, accelerating the development of new immunotherapies and optimizing their clinical use.

Ultimately, the integration of advanced technologies and multi-disciplinary approaches, especially those focused on immune responses, will solidify organoid models as indispensable tools in both preclinical research and clinical decision-making, driving the future of precision oncology and immunotherapy [145, 147].

## Conclusion

Organoid models are crucial tools in cancer immunotherapy, providing a more accurate simulation of the TME and enhancing the evaluation of immune therapies like ICIs, CAR-T cells, and tumour vaccines. By integrating immune and stromal components, these models help bridge the gap between preclinical and clinical research. Despite their potential, challenges remain, including the need for improved immune system simulation and long-term drug sensitivity testing. Emerging technologies, such as microfluidic platforms, 3D bioprinting, and genetically engineered organoids, are addressing these limitations, while AI integration further optimizes predictive accuracy and treatment strategies. Moving forward, these advancements will strengthen the role of organoid models in personalized cancer treatment, ultimately improving the efficacy of immunotherapies and patient outcomes.

## Data Availability

Data sharing not applicable to this article as no datasets were generated or analysed during the current study.
